# Comparison of Ki67 index and P16 expression in different grades of cervical squamous intraepithelial lesions

**DOI:** 10.22088/cjim.14.1.69

**Published:** 2023

**Authors:** Maryam Sadat Hosseini, Maryam Talayeh, Noushin Afshar Moghaddam, Maliheh Arab, Farah Farzaneh, Tahereh Ashrafganjoei

**Affiliations:** 1 Preventative Gynecology Research Center, Department of Obstetrics and Gynecology, Imam Hossein Hospital, Shahid Beheshti University of Medical Sciences, Tehran, Iran; 2 Department of Gynaeco-oncology, Imam Hossein Medical Center, Shahid Beheshti University of Medical, Tehran, Iran; 3 Department of Pathology, Imam Hossein Medical Center, Shahid Beheshti University of Medical Sciences, Tehran, Iran

**Keywords:** Ki 67 index, P16, Cervical Intraepithelial Neoplasia, High Grade Squamous Intraepithelial Lesion, Immunohistochemistry

## Abstract

**Background:**

the assessment of P16 expression and Ki-67 proliferative index is now proposed as an adjunct test for the diagnosis of high-risk precursor lesions for cervical cancer. The aim of the present study was to elucidate the quality expression of P16 and quantification Ki-67 index in different types of cervical intraepithelial neoplasia and also to determine the cutoff for Ki67 index to predict the severity of CIN lesions.

**Methods:**

This cross-sectional study was conducted on patients with colposcopic indication. Selected samples with different CIN grades were examined for P16 and Ki-67 index by immunohistochemical (IHC) methods.

**Results:**

All LSIL (CIN I) cases were negative for P16, while in 58.7% of HSIL cases (CIN 2/3), P16 was positive. The mean Ki67 index in the present study was 3.13 ± 2.65 in the upper two/third of the squamous epithelium in the LSIL group and 19.04 ±36.40 in the HSIL group, which was statistically significant. Also, the mean Ki67 index in full thickness squamous epithelium in HSIL group was significantly higher than LSIL. The sensitivity of P16 and Ki67 index in our study was 58.73%, 66.67% and the specificity was 100% and 100%, respectively.

**Conclusion:**

Assessment of P16 expression and Ki67 index can be used to distinguish low grade (CIN1) intraepithelial lesion from high grade (CIN2/3) intraepithelial or precancerous lesions.

Cervical cancer is the second most common malignancy in women worldwide (1), which originates from cervical intraepithelial neoplasia (CIN). The World Health Organization (WHO) latest classification system applied low-grade squamous intraepithelial lesions (LSIL) and high-grade squamous intraepithelial lesions (HSIL) (2). However, CIN was previously categorized into CIN1, CIN2, and CIN3 based on the degree of epithelial involvement. CIN can be effectively treated to prevent its progressing to cervical cancer. CIN1 is usually not precancerous and does not require treatment, but close follow-up is recommended, while the rate of progression of CIN2/3 to invasive cervical cancer is about 10 to 40% (3). Given the malignant potential of CIN2/3, it is important to have an accurate CIN rating and proper treatment of these patients (4). CIN2 and CIN3 are usually treated with excisional methods such as cold knife conization or Leep (5). Histological diagnosis based on cervical biopsy is often considered the "gold standard" for the diagnosis of cervical dysplasia. However, there is significant inter/intra observer variability, which makes it difficult to diagnose non-neoplastic lesions as well as to differentiate CIN1 from CIN2/3 that may lead to over or inadequate treatment (6). 

Therefore, accurate diagnosis of cervical lesions is important for decision making and treatment of patients. It takes 5 to 15 years for cervical intraepithelial neoplastic lesions to progress to invasive lesions (7). In extensive epidemiological and molecular biology studies, human papillomavirus (HPV) infection has been identified as the leading cause of cervical cancer (8). Stable infection with high-risk HPV is associated with cervical cancer. HPV induces cervical cancer through the uncontrolled G1-S cycle (9). High-risk HPV E6 and E7 proteins inhibit p53 and pRb proteins, which regulate the cell cycle in G1-S transport. P16INK4a (P16) is a protein belonging to cyclin-dependent kinase (CDK) 4 inhibitors (10). By interacting with CDK4 and P16, CDK6 inhibits the formation of the cyclin D/CDK4,6 complex, which is a protein that stimulates cell proliferation (11). In other words, CDK4,6 which is a checkpoint in phase G1 to phase S, is inhibited by P16 (11). Overexpression of P16 is also associated with functional inactivation of retinoblastoma protein (Rb) which is regulated by HPV E6 and E7 proteins, which commonly occurs in HPV infection (12). 

P16 expression is usually low in healthy cells. Immunohistochemical staining of P16 has been widely used as a biomarker of cervical cancer in the vast majority of high-risk HPV cases with severe CIN lesions (13). However, some normal cervical tissues express P16, and a small number of cases of CIN2 or higher cause poor or negative P16 staining, so the use of P16 staining limits the diagnosis of cervical lesions (14). For this reason, another biomarker called Ki67 can be used to more accurately diagnose lesions (15). The Ki-67 protein in the nucleus is a proliferative marker that is expressed in stages G1, S, G2 and M of the cell cycle (16). Analysis of Ki-67 expression in histological samples from women with CIN1 or CIN2 has shown that Ki-67 index is an independent predictor of CIN grade of intraepithelial lesion (17). As a result, ki-67 index can be expressed during cell proliferation resulting from HPV infection. High expression of Ki-67 index is associated with the severity of cervical lesions but is not associated with HPV infection (18). Therefore, Ki-67 index expression assessment with P16 staining has been proposed as an adjunct test for the diagnosis of high-risk precursor lesions or cervical cancers (19). The aim of the present study was to elucidate the quantitative immunohistochemical patterns of Ki-67 index and quality expression of p16 in CIN types and also to determine the cutoff for Ki67 index to predict the severity of CIN lesions.

## Methods

This study was a cross sectional study. All patients aged 21 to 54 years who referred to the colposcopic clinic of Imam Hossein Hospital between 2019 and 2021 with colposcopic indication (abnormal cervical cytology and vaginal discharge resistant to treatment, post coital bleeding) were included in the study. All patients underwent biopsy with abnormal colposcopy. The samples were examined by immunohistochemistry technique specific for Ki67 index and quality expression of P16. Insufficient specimens (lack of covering squamous epithelium to assess squamous cell dysplasia), diagnostic discrepancies between two academic pathologists, ulcerated specimens (presence of ulcer or erosion in superficial epithelium), specimens with severe inflammatory response or with the reparative changes, the samples of postmenopausal women and the samples with improper fixation or processing were all excluded from the study.

**Figure 1:**
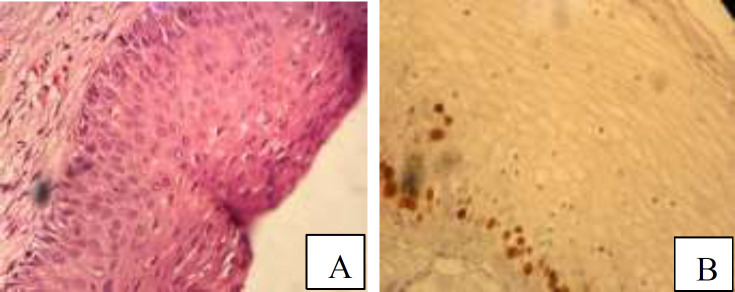
A: Photomicrograph of low grade squamous intraepithelial lesion (CIN1) revealed decreased cellular maturation, nuclear enlargement and mild irregularity in lower part of epithelium (H&E staining, ×200). B: Immunostaining for Ki67 in low grade squamous intraepithelial lesion (CIN1) showed low proliferative activity especially absent in upper part of epithelium

**Figure 2:**
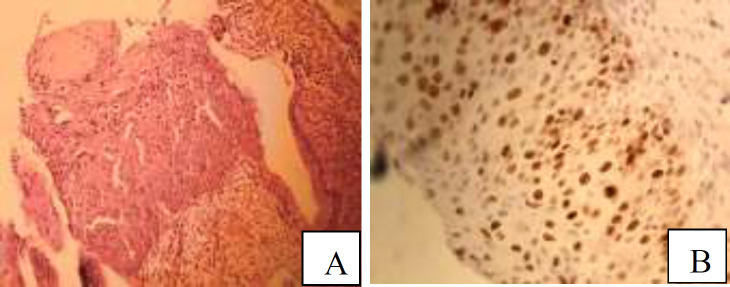
A: Photomicrograph of high grade squamous intraepithelial lesion (CIN3) revealed decreased cellular maturation, nuclear enlargement, hyperchromasia and irregularity in full thickness of epithelium (H&E staining, ×200). B: Immunostaining for Ki67 in high grade squamous intraepithelial lesion (CIN3) showed high proliferative activity in full thickness and also in upper part of epithelium

**Figure 3:**
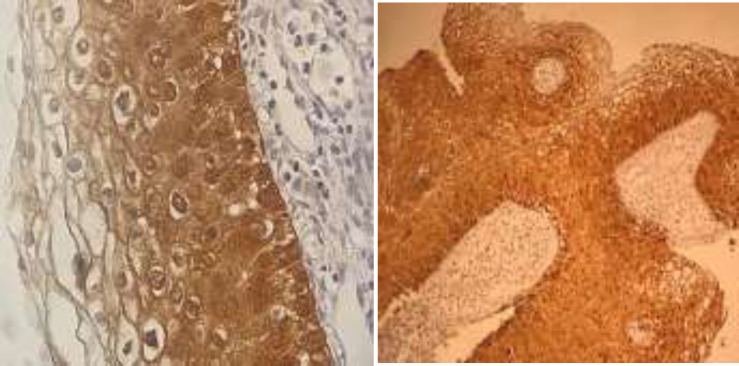
CIN3/diffuse block staining p16

Selected samples with different CIN grades of intraepithelial lesions were examined for P16 (JC2 clone, Mouse monoclonal Antibody, Pleasanton CA, USA) and Ki-67 index for IHC evaluation. In the first step, a 3 μm thin section was obtained from the selected blocks, and then the samples were deparaffinized and hydrated. Antigen exposure against 1% citrate buffer (PH=6) was performed in the microwave for 20 minutes. Slides were incubated with Human anti Ki67 antibody, SP6 clone, Cat. No.: BRB040, Germany) at room temperature. Epithelial cells with positive nuclear staining were selected for counting. For Ki-67 immunohistochemistry, the product was a color reaction (brown) at the antigen site in the cell nucleus. At least 8 hotspots previously selected at low magnification were selected and the percentage of Ki-67 positive cells (Ki-67 index or proliferative index) was obtained. Ki67 index was evaluated in the upper two thirds and full thickness of the squamous epithelium (figure 1, 2). IHC results for P16 expression should be considered positive in diffuse block staining pattern (figure 3).

Quantitative data were displayed using mean and standard deviation and qualitative data were displayed using frequency and percentage. Chi-square and analysis of variance (and Gabriel test as post hoc test) were used to compare the quantities between the study groups. The values of sensitivity, specificity, positive and negative predictive values and the area under the ROC curve were used to evaluate the diagnostic value of biomarkers. R software version 3.6.1 was used for data analysis. Significance level was considered 0.05 for statistical tests. The study protocol was verified by the ethical committee of Shahid Beheshti University of Medical Sciences (IR.SBMU.RETECH.REC.1399.1102).

## Results

The results according to LSIL and HSIL categorization. Overall, 126 patients were studied that of those, 50% were categorized as the LSIL or CIN I group and 50% as the HSIL group (26.2% in CIN II and 23.8% in CIN III subgroups). The mean age of patients was 35.94±7.62 years ranged 24 to 53 years. As shown in Table 1, no difference was revealed in the mean age (p= 0.328) between the two groups suffering HSIL and LSIL, however abnormal pap smear was more prevalent in the HSIL group as compared to the LSIL group (p <0.001). The subtype of HPV 16 was notably more prevalent in HSIL than in LSIL group; however there was no difference in HPV 18 or other subtypes between the two groups. Regarding Pap smear results, the main findings for low risk group included atypical Squamous Cells of Undetermined Significance (ASC-US) reporting in 17.5% followed by LSIL in 9.5%, but the most common feature in high risk group was HSIL (23.8%) followed by high-grade squamous intraepithelial lesions (ASC-H) (12.7%), LSIL (12.7%), ASC-US (11.1%) and Atypical glandular cell (AGC) (1.6%).

The mean Ki67 index in the upper two-third of squamous epithelium was 2.65±3.13 in the group with low risk diagnosis (CIN I) and 19.04±36.40 in the group with high risk diagnosis (HSIL), which was significantly higher in the HSIL group than another low risk group (p<0.001). Also, while none of the patients with CIN I diagnosis had P16, 58.7% of patients with high-risk diagnosis had P16 (p<0.001) (Table 1). 

**Table 1:** Patient’s characteristics and pathological markers according to study groups

**Variables**	**LSIL (CIN I)**	**HSIL**	**P value**
Mean age, year	35.27±8.35	36.60±6.83	0.328
Abnormal pap smear, N (%)	17 (27)	37 (58.7)	<0.001
HPV 16, N (%)	3 (4.8)	16 (25.4)	0.001
HPV 18, N (%)	3 (4.8)	3 (4.8)	0.999
Other HPV, N (%)	26 (41.3)	19 (30.2)	0.432
Pathological markers			
Ki67 (Upper 2/3 mean±SD)	2.65±3.13	36.40±19.04	<0.001
Ki67 (Full thickness mean±SD)	26.41±11.09	67.25±19.45	<0.001
P16 N (%)	0 (0)	37 (58.7)	<0.001

**Figure 4:**
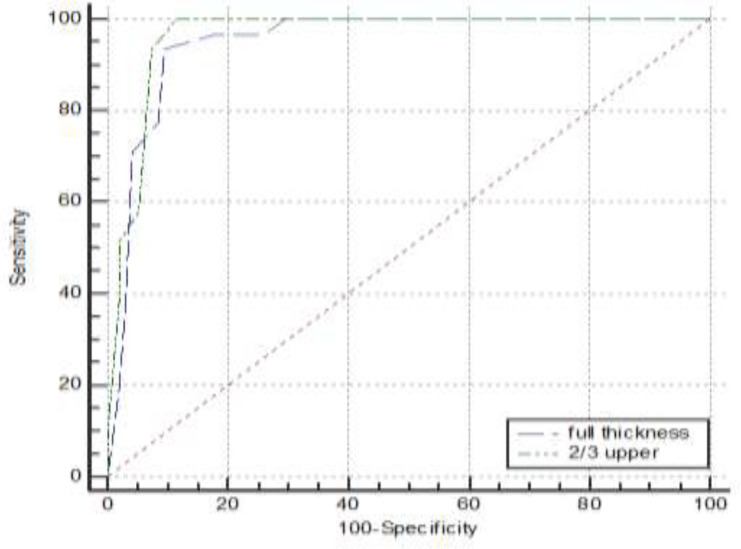
Receiver operating characteristics (ROC) curve of upper 2/3 and full thickness for diagnosis of low grade from high grade CIN

According to the ROC curve analysis (Table 2, Figure 4), the area under the curve for predicting high risk diagnosis (HSIL) for full thickness parameter was 0.949 (95%CI: 0.895 to 0.980) and for upper 2/3 was 0.963 (95%CI: 0.914 to 0.989) indicating high value of these indices for discriminating high risk from low-risk subgroups. In this regard, the best cutoff value for upper 2/3 was 20.0 (yielding a sensitivity of 66.7% and a specificity of 100%) and for full thickness was 60.0 (yielding a sensitivity of 32.0% and a specificity of 100%). Also, the sensitivity and specificity of P16 for predicting high risk condition were 58.7% and 100% respectively (Table 2). 

**Table 2:** Diagnostic accuracy of markers for prediction of pathology result

**Marker**	**Cut-off point**	**Sensitivity (95% CI)**	**Specificity (95%CI)**	**PPV** **(95%CI)**	**NPV** **(95%CI)**
Ki67 (Upper 2/3)	>20	66.67% (53.66% - 78.05%)	100% (94.31% - 100%)	100% (91.40% - 100%)	75% (64.36% - 83.81%)
Ki67 (Full thickness)	>60	60.32% (47.20% - 72.43%)	100% (94.31% - 100%)	100% (90.75% - 100%)	71.59% (60.98% - 80.70%)
P16	---	58.73% (45.62% - 70.99%)	100% (94.31% - 100%)	100% (90.51% - 100%)	72.79% (60.19% - 79.95%)

## Discussion

Accurate histological grading of cervical intraepithelial neoplasia (CIN) is clinically important because CIN2 and CIN3 are considered precursors of invasive cervical carcinoma and treatment is indicated. Histopathology is a gold standard for diagnosing CIN. However, inter and intra observer variability is relatively high in the interpretation of cervical biopsy specimens. Therefore, the distinction between CIN1 and CIN2/3 is still challenging. On the other hand, accurate diagnosis of cervical lesions is important for decision making and treatment of patients, and having an alternative method and diagnostic assistance to differentiate these lesions is reasonable and useful. In this study, 126 patients were studied. In the present study, all LSIL (CIN I) cases were negative for P16, while in 58.7% of HSIL cases (CIN 3.2), P16 was positive. Also, the sensitivity and specificity of P16 for differentiating HSIL from LSIL lesions were 58.73% and 100%, respectively, indicating the high specificity of the test. The expression level of strong block diffuse P16 in different studies is in the range of 8.3% to 67.3% for CIN I, 33.3% to 100% for CIN II, and 73.8% to 100% for CIN III (Table 3). In this regard, with the progression of CIN lesions, the expression of P16 also increases, which is consistent with the present study. The low expression of P16 in CIN II (27.3%) compared to other studies could be due to differences in the positive expression criteria of P16. Thus, in studies that considered positive staining only in the nucleus or cytoplasm, the expression level of P16 was higher than that in other studies, including our study, which considered positive staining of the nucleus and cytoplasm together. It should also be noted that one of the reasons for reporting positive P16 cases in CIN I in some studies may be due to underestimation of CIN II cases from the beginning. One possible reason for the lower expression of P16 in LSIL lesions may be the presence of low-risk HPV types (approximately 20% LSIL is negative for high-risk HPV types). Because the tendency for the low-risk HPV E7 protein for Rb is much lower than for high-risk HPVs, there will be no overexpression of P16. In addition, Keating et al in their study showed that low-risk HPV was associated with lower P16 expression, and that different stages of HPV-induced cervical neoplasia may have different levels of P16 expression. Additionally, a number of studies have concluded that CIN I cases with a P16 positive are more likely to progress to CIN II/III.

The mean Ki67 index in the present study was 3.13 ± 2.65 in the upper two/third of the squamous epithelium in the LSIL group and 19.04±36.40 in the HSIL group, which was statistically significant. Also, the mean Ki67 index in full thickness squamous epithelium in HSIL group was significantly higher than LSIL. In assessment of various studies, the rate of Ki67 index positive cases in the upper two/third of the squamous epithelium in CIN I was 18 to 41%, in CIN II was 55 to 95%, in CIN III was 73.8% to 95% (Table 4). With the progression of CIN lesions, the expression of Ki67 index increases, which is consistent with the results of the present study. In previous studies, the cut off for the Ki67 index to differentiate HSIL from LSIL has not been studied and therefore the results of the present study have been unique in this respect. The sensitivity of P16 and Ki67 index in our study was 58.73%, 66.67% and the specificity was 100% and 100%, respectively, which lower sensitivity and higher specificity had compared to other studies (Table 5). According to the results of various studies, the sensitivity and specificity of Ki67 and P16 biomarkers in predicting the grading of cervical intra epithelial neoplasia (CIN) lesions were higher than the HPV test. It is suggested that in future studies, computer software be used to quantify ki67 index because manual quantitative counting of the Ki 67 index can be influenced by factors such as the thickness of the sections and inter and intra observer variability. It is recommended that a study be conducted in collaboration with computer engineers to design artificial intelligence to quantify Ki 67 index. Also, it is better ki-67 and p16 compared with high risk HPV testing in future studies.

In conclusion the abnormal P16 expression (block staining pattern) and high Ki67 index in upper part of squamous epithelium should be considered as useful diagnostic tool for differentiation LSIL and HSIL. Our study showed that in case of doubt in the CIN grading, the use of Ki67 index to distinguish between CIN1 and CIN2/3 would be useful.

**Table 3:** Evaluation of P16 staining in different grades of CIN in different studies:

**Author**	**P16 Negative**			**Strong positive P16**			**P-value**
	**CIN I**	**CIN II**	**CIN III**	**CIN I**	**CIN II**	**CIN III**	
Hosseini (20)				0%	27.3%	93.3%	<0.001
Mandal (21)				33.3%	58.1%	73.8%	
Xing (22)				24.4%	87.5%		
Hebbar (23)	40%	20%	10%	50%	70%	90%	
Kanthiya (24)				10.4%	78.7%		
Zhong (25)	52.81%	7.38%	3.06%	67.32%	98.85%	99.38%	<0.001
Aslani (26)				50%	100%	100%	
Nam (27)	91.6%	66.7%		8.3%	33.3%	100%	
Agoff (28)				38%	64%	86%	

**Table 4:** Evaluation of Ki67 staining in different degrees of CIN in different studies:

**Author**	**ki67 Negative**			**Ki67 Positive**			**P-value**	
	**CIN I**	**CIN II**	**CIN III**	**CIN I**	**CIN II**	**CIN III**		
Mandal (21)				33.3%	58.1%	73.8%		
Xing (22)				35.6%	95%			
Hebbar (23)	60%	20%		40%	80%	90%		
Kanthiya (24)				22.6%	75.4%	75.4%		
Zhong (25)	79.65%	23.8%	6.74%	19%	75%	93.25%		<0.001
Nam (27)	58.3%	16.7%	8.3%	41.7%	83.3%	91.7%		
Agoff. (28)				18%	55%	76%		

**Table 5:** Evaluation of sensitivity and specificity of P16 and Ki67 in the diagnosis of CIN lesions

**Author**	**P16**					**Ki67** **)** **2/3 upper)**			
	**sensitivity**	**specificity**	**PPV**	**NPV**	**sensitivity**		**specificity**	**PPV**	**NPV**
Hosseini (21)	58.73%	100%	100%	72.79%	66.67%		100%	100%	75%
Xing (23)	87.5%	75.65	76.1%		95%		64.4%	70.3%	
Hebbar (23)	76.2%	87.5%	96.9%	41%	90.5%		87.5%	95%	60%
Kanthiya (24)	90.5%	84.5%			82.1%		88.6%		
Aslani (26)	91.3%	98.1%	95.4%	96.3%	95.6%		85.1%	91.3%	98.1%
